# A nanobody against the V-ATPase c subunit inhibits metastasis of 4T1-12B breast tumor cells to lung in mice

**DOI:** 10.18632/oncotarget.28638

**Published:** 2024-08-14

**Authors:** Zhen Li, Mohammed A. Alshagawi, Rebecca A. Oot, Mariam K. Alamoudi, Kevin Su, Wenhui Li, Michael P. Collins, Stephan Wilkens, Michael Forgac

**Affiliations:** ^1^Department of Developmental, Molecular, and Chemical Biology, Tufts University School of Medicine, Boston, MA 02111, USA; ^2^Program in Pharmacology and Drug Development, Graduate School of Biomedical Sciences, Tufts University, Boston, MA 02111, USA; ^3^Program in Cellular, Molecular and Developmental Biology, Graduate School of Biomedical Sciences, Tufts University, Boston, MA 02111, USA; ^4^Department of Cancer Immunology and Virology, Dana Farber Cancer Institute, Harvard Medical School, Boston, MA 02215, USA; ^5^Department of Pharmacology, University of Minnesota School of Medicine, MN 55455, USA; ^6^Department of Pharmacology, College of Pharmacy, Prince Sattam Bin Abdulaziz University, Al-Kharj 11942, Saudi Arabia; ^7^Korro Bio, Cambridge, MA 02139, USA; ^8^Department of Biochemistry and Molecular Biology, SUNY Upstate Medical University, Syracuse, NY 13210, USA; ^9^Foghorn Therapeutics, Cambridge, MA 02139, USA; ^*^These authors contributed equally to this work

**Keywords:** vacuolar ATPase, breast cancer, invasion, tumor metastasis, tumor growth

## Abstract

The vacuolar H^+^-ATPase (V-ATPase) is an ATP-dependent proton pump that functions to control the pH of intracellular compartments as well as to transport protons across the plasma membrane of various cell types, including cancer cells. We have previously shown that selective inhibition of plasma membrane V-ATPases in breast tumor cells inhibits the invasion of these cells *in vitro*. We have now developed a nanobody directed against an extracellular epitope of the mouse V-ATPase c subunit. We show that treatment of 4T1-12B mouse breast cancer cells with this nanobody inhibits V-ATPase-dependent acidification of the media and invasion of these cells *in vitro*. We further find that injection of this nanobody into mice implanted with 4T1-12B cells orthotopically in the mammary fat pad inhibits metastasis of tumor cells to lung. These results suggest that plasma membrane V-ATPases represent a novel therapeutic target to limit breast cancer metastasis.

## INTRODUCTION

Breast cancer is one of the most diagnosed cancers, accounting for almost one-third (30%) of all new diagnoses in women in 2022 [1]. At time of diagnosis, 20–30% of patients with early-stage breast cancer will go on to develop metastatic breast cancer, and 6–10% of all patients with breast cancer have stage IV disease at time of diagnosis (called *de novo* metastatic breast cancer) [2]. The development of metastases is commonly associated with a poor prognosis compared to non-metastatic breast cancer, with a median survival time of 2–3 years [2]. The most common sites of distant metastasis in breast cancer include: bone, liver, lung, and brain [2]. Although metastasis is the leading cause of death from cancer there are currently no effective therapies to prevent metastasis [3].

Metastasis is a multistep, complex process that involves the intravasation of tumor cells from the primary tumor site into the circulation or lymphatic system, and extravasation of cells into secondary sites throughout the body [4–6]. In order for tumor cells to metastasize, they have to acquire an invasive phenotype, which allows them to penetrate and degrade the basement membrane and extracellular matrix [4–6]. This phenotype is enhanced by an acidic extracellular pH that increases the activity of secreted acid-dependent proteases (cathepsins) that participate in degradation of ECM, thus promoting tumor cell invasiveness [7]. The activity of the vacuolar H^+^-ATPase plays an important role in control of extracellular pH [8]. The V-ATPase is an ATP-dependent proton pump that is present in both intracellular membranes as well as the plasma membrane of specialized cell types in eukaryotes [8]. The V-ATPase consists of a peripheral V1 domain, that is responsible for ATP hydrolysis and contains subunits A–H, as well as a membrane-embedded V0 domain, that carries out proton transport and is composed of subunits a, c, c″, d, e and f [8–11]. Intracellular targeting of V-ATPases is controlled by isoforms of subunit a, with a3 and a4 targeting the V-ATPase to the plasma membrane of osteoclasts and renal intercalated cells, respectively [8].

It has been shown that the V-ATPase plays an important role in promoting the invasiveness of many cancer cell types, including breast cancer cells [9]. Specific inhibitors of the V-ATPase (bafilomycin and concanamycin) inhibit the *in vitro* invasiveness of several breast cancer cell lines [12–16]. This has also been observed in prostate cancer cells [17]. Moreover, highly invasive breast cancer cells express higher levels of isoforms of the a subunit (a3 and a4) that target V-ATPases to the plasma membrane [13, 14, 16]. Selective inhibition of a3 and a4-containing V-ATPases using isoform-specific siRNA inhibits both the invasiveness of highly metastatic breast cancer cell lines and the expression of V-ATPases at the cell surface [13–16]. Importantly, overexpression of a3 in non-invasive breast cancer cells increases their invasiveness and plasma membrane expression of the V-ATPase [14]. These results suggest that breast cancer cells upregulate expression of a3 or a4 resulting in increased targeting of V-ATPases to the cell surface where they promote tumor cell invasion. Significantly, samples of human breast cancer show much higher levels of expression of a3 at the RNA level relative to normal tissue, and expression is highest in invasive breast carcinoma compared to non-invasive solid tumors and normal breast tissue [15].

We have previously shown that selective inhibition of plasma membrane V-ATPases using either a membrane impermeant V-ATPase inhibitor or an antibody directed against an epitope tag introduced into the extracellular domain of the c subunit is able to inhibit both proton transport across the plasma membrane and the invasiveness of MDA-MB231 cells *in vitro* [18]. This study provided a proof of principle that specific inhibition of cell surface V-ATPases was sufficient to inhibit tumor cell invasion. This is important as the available V-ATPase inhibitors (including bafilomycin and concanamycin) are membrane permeant and therefore inhibit all the V-ATPases in the cell, including those in the endocytic and secretory systems, resulting in potentially toxic inhibition of endocytosis, neurotransmitter uptake and other essential processes that depend upon the activity of intracellular V-ATPases [8, 10, 19].

**Figure 1:**
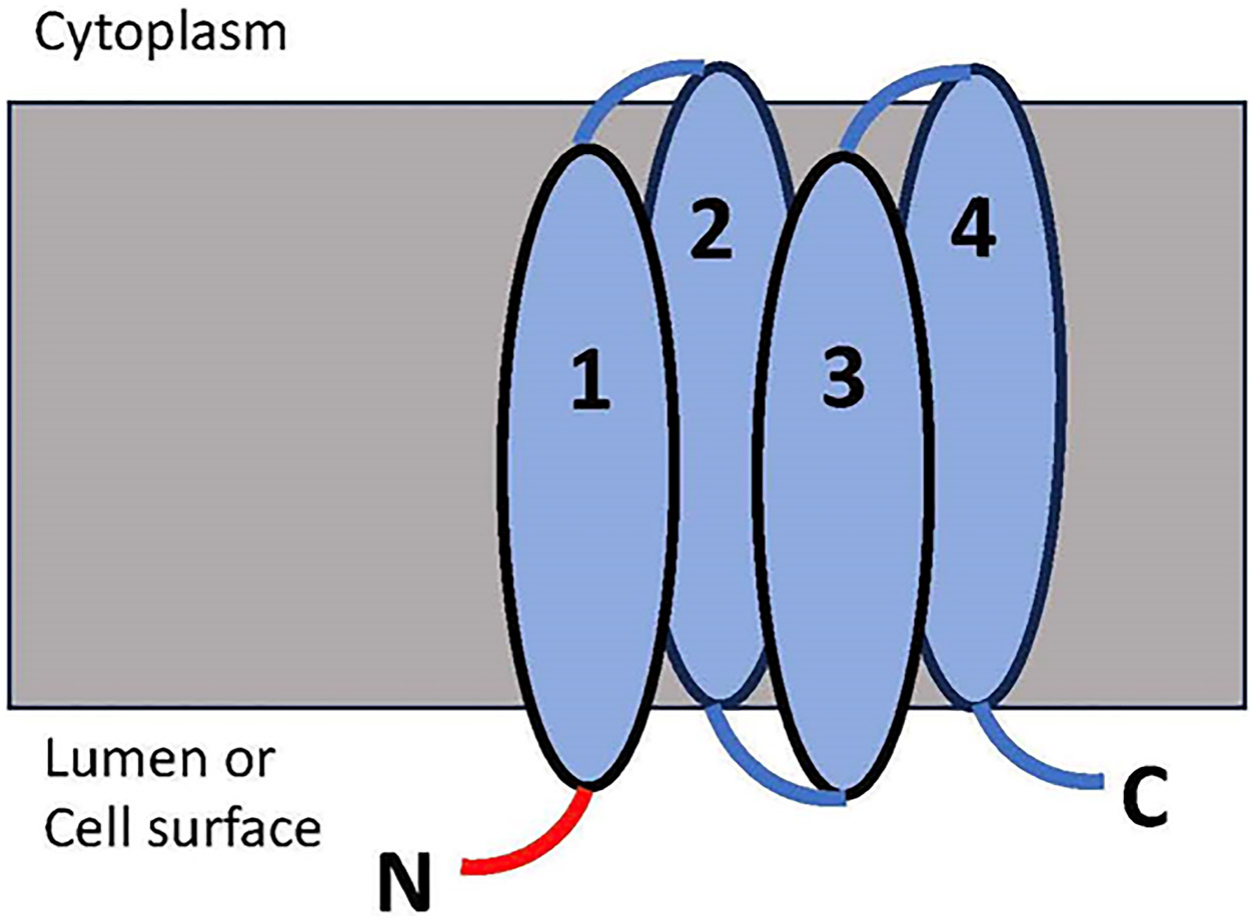
The membrane topology of the V-ATPase subunit c targeted by the prepared nanobody. There are three regions present on the extracellular side of the c subunit, with the N-terminal tail (N, in red), C-terminal tail (C) and the TM2/3 loop. The N-terminal tail has the greatest extension from the membrane; thus this region was chosen for the preparation of the nanobody.

In the present study we describe the development of an inhibitory nanobody directed against an extracellular epitope present in the native V-ATPase c subunit. Since the N-terminal of subunit c displays the greatest degree of extension from the membrane surface on the non-cytoplasmic side of the protein based on recent cryo-EM structures of the rat and bovine V-ATPases [20, 21], our antibody was designed against this N-terminal tail (Figure 1). In mammals, since subunit c is generally well conserved in overall sequence, there would be low immunogenicity of produced peptide antigens using traditional animal methods for antibody generation [22]. In order to avoid the requirement of animal immunization, nanobody phage display was used to raise single domain antibodies *in vitro*. This approach allows us to identify and isolate antibody sequences that would have been suppressed in an animal model due to selection against antibodies that recognize self-antigens. We have now characterized such an anti-V-ATPase c subunit nanobody in terms of its inhibitory effect on V-ATPase-dependent proton transport in 4T1-12B mouse breast cancer cells, the *in vitro* invasion of these cells and the growth and metastasis of breast tumors in mice following implantation of these cells in the mouse mammary fat pad. Our results suggest that anti-V-ATPase antibodies directed against an extracellular epitope of the V-ATPase are capable of inhibiting activity at the surface of cancer cells as well as *in vitro* invasion and *in vivo* metastasis of these cells in a mouse model, a finding that represents an exciting step towards a new therapy for limiting breast cancer metastasis.

## RESULTS

### A synthetic nanobody targeting the mouse V-ATPase subunit c inhibits extracellular acidification by 4T1-12B mouse breast cancer cells

**Figure 2:**
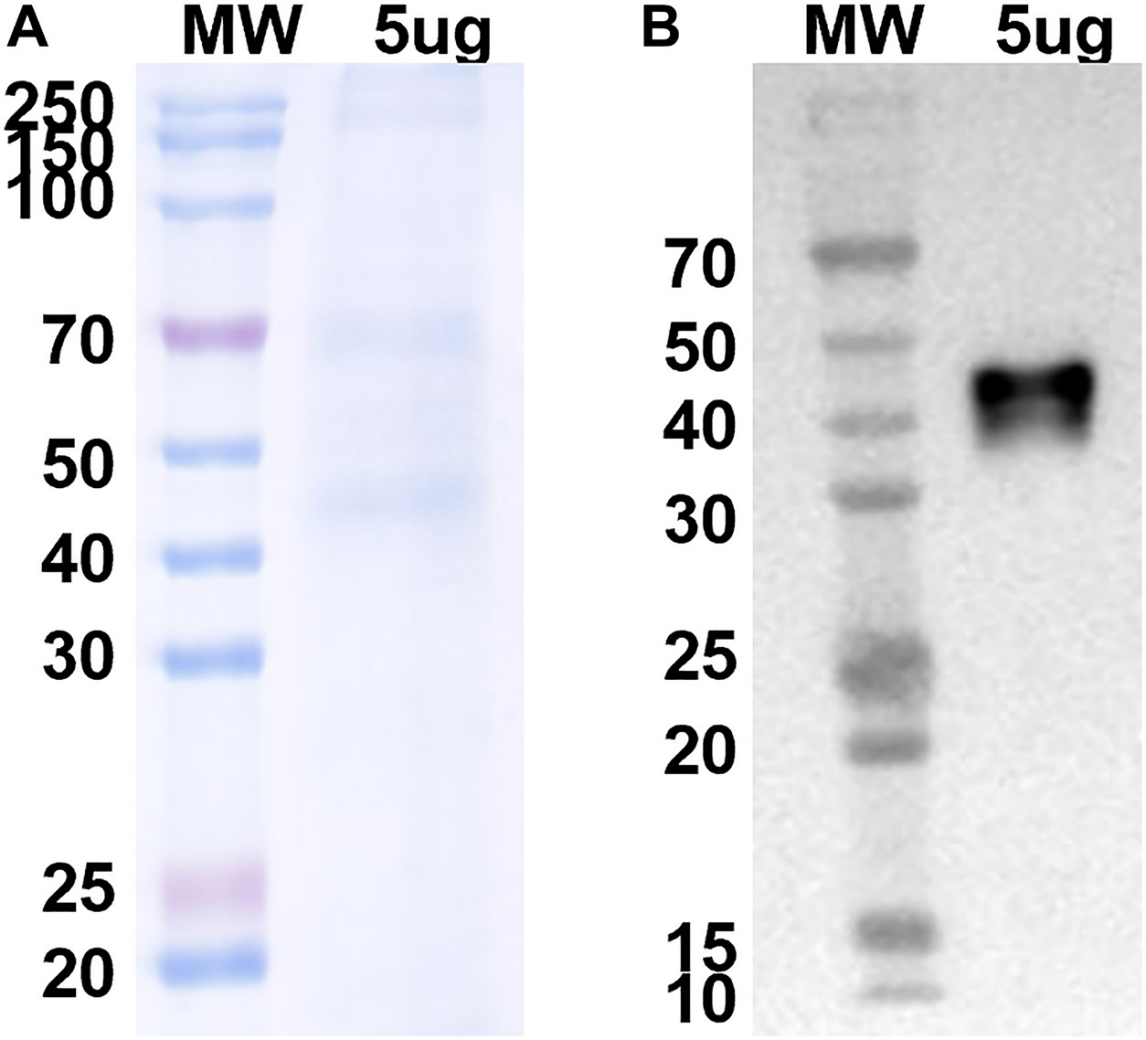
Coomassie blue stained gel and Western blot analysis using anti-His antibody of purified His-tagged anti-V-ATPase nanobody. 5 μg of Ni affinity-purified nanobody was applied to SDS-PAGE under non-reducing conditions (**A**) and proteins were transferred to nitrocellulose followed by Western blotting using an anti-His antibody and ECL (**B**). Data were provided by Proteogenix.

A camelid nanobody directed against the N-terminus of the mouse V-ATPase c subunit was prepared (epitope is shown in Figure 1) as described under Experimental Procedures. In nanobody phage display, antigens are screened against a bacteriophage library where diverse (>1 × 10^6^) sequences of camelid single chain variable heavy domains are fused to the phage coat protein. The single chain nanobodies have been fused to a linker sequence and the resulting chimeras dimerized by disulfide bonding to create a bivalent molecule similar to a traditional antibody. Figure 2 shows a Coomassie blue stained gel and Western blot of the Ni affinity-purified anti-V-ATPase nanobody as detected using an anti-His antibody. The molecular weight of the monomeric form of the nanobody is predicted from the sequence to be 28.4 kDa while the disulfide bonded dimer is predicted to have a molecular weight of 56.8 kDa. It should be noted that although SDS-PAGE was performed under non-reducing conditions, the apparent molecular weight of the His-reactive band is approximately 45 kDa, suggesting that the disulfide bonded form migrates somewhat faster than the predicted molecular weight.

**Figure 3:**
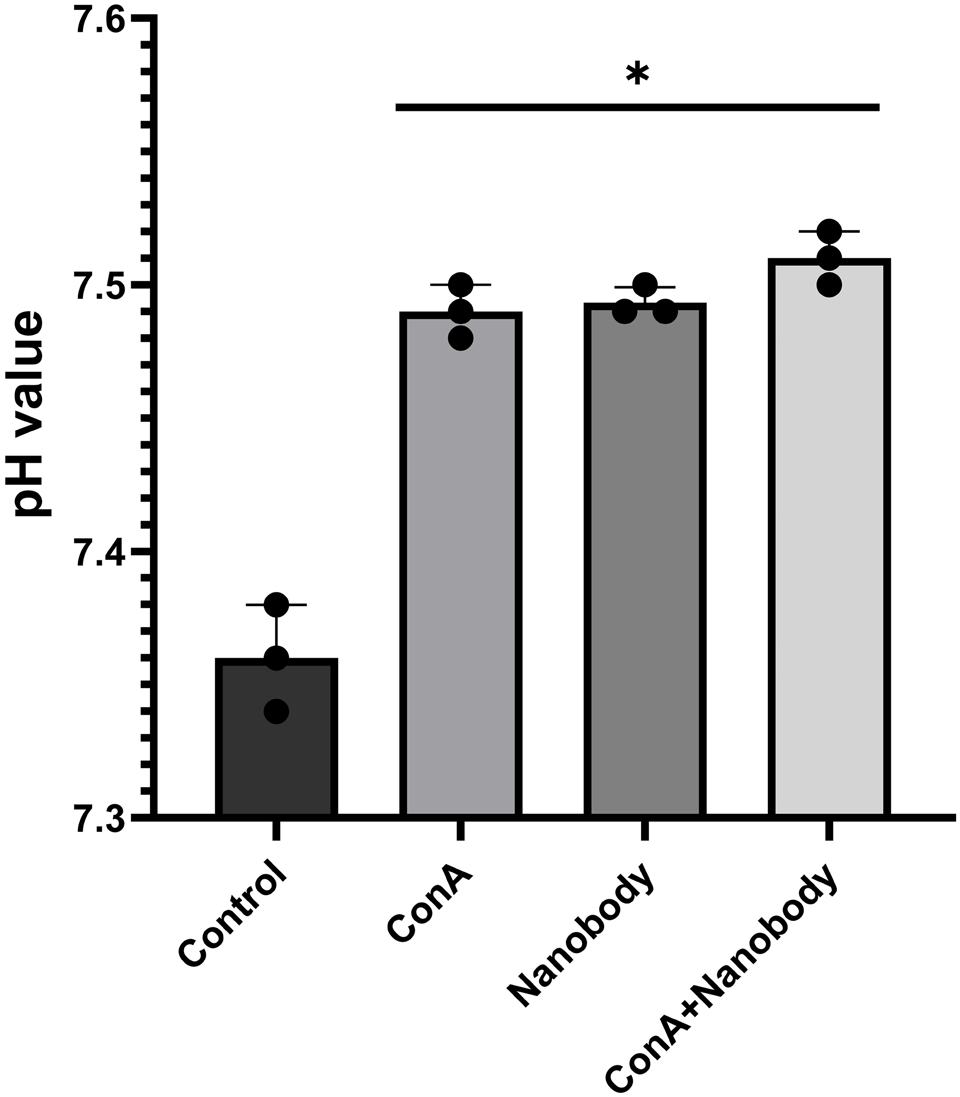
Treatment of 4T1-12B cells with anti-V-ATPase nanobody inhibits extracellular acidification to the same extent as concanamycin. 4T1-12B cells were suspended in DMEM containing 1:5 diluted PBS (control, conA) or 1:5 diluted PBS containing 66 μg nanobody (final concentration 1.17 μM) (nanobody, conA plus nanobody) and incubated for 2 hrs at 37 C. Cells were then washed and resuspended in unbuffered DMEM containing 1:5 diluted PBS plus 0.1% DMSO (control), 1:5 diluted PBS plus 10 nM conA (conA), 1:5 diluted PBS containing 1.17 μM nanobody plus 0.1% DMSO (nanobody) or 1:5 diluted PBS containing 1.17 μM nanobody plus 10 nM conA (conA + nanobody). Where indicated, PBS was diluted 1:5 with DMEM. Following incubation for 2 hrs at 37 C, the media was removed and the pH measured using a pH meter. Shown are results for 3 wells from one representative trial, error bars are SEM, ^*^ *p* < 0.05 for each condition relative to control.

We wished to determine whether this nanobody was able to inhibit V-ATPase-dependent acidification of the media in which mouse 4T1-12B cells were suspended. 1 × 10^5^ cells were suspended in Dulbecco’s modified Eagle media to which was added either PBS or nanobody (66 μg) in PBS (final nanobody concentration of 1.17 μM) followed by incubation for 2 hrs at 37^o^C to allow time for the nanobody to bind to its cell surface target. The media was then replaced with unbuffered DMEM containing either 0.1% DMSO plus PBS (control), PBS plus concanamycin (10 nM) in DMSO or 0.1% DMSO plus PBS containing 66 μg nanobody (final concentration of 1.17 μM) followed by incubation for an additional 2 hrs at 37°C. The media was then removed and the pH was measured using a pH meter, with each measurement done in triplicate. As can be seen in Figure 3, treatment of cells with the nanobody led to the same increase in extracellular pH as treatment with concanamycin. To determine whether the nanobody and concanamycin were affecting the extracellular pH by inhibiting the same target, the effect of treatment with both concanamycin plus nanobody was compared with treatment with either concanamycin or nanobody alone. As can be seen in Figure 3, treatment with both concanamycin and nanobody increased the extracellular pH by nearly the same amount as either treatment alone. The increase in extracellular pH for nanobody treatment alone was 0.11 (±0.01) (*n* = 5 independent trials), for conA treatment alone was 0.11 (±0.01) (*n* = 4 independent trials) and for the combination treatment 0.15 (±0.01) (*n* = 3 independent trials). No effect of treatment with nanobody on cell viability, as measured by Trypan blue exclusion, was observed. One possible explanation for the slightly greater increase in extracellular pH with the combined treatment may be that either treatment alone may not be completely effective at inhibiting cell surface V-ATPases. These results indicate that treatment of cells with the nanobody leads to inhibition of V-ATPase-dependent extracellular acidification.

### Treatment of cells with the anti-V-ATPase nanobody inhibits *in vitro* invasion by 4T1-12 B cells

**Figure 4:**
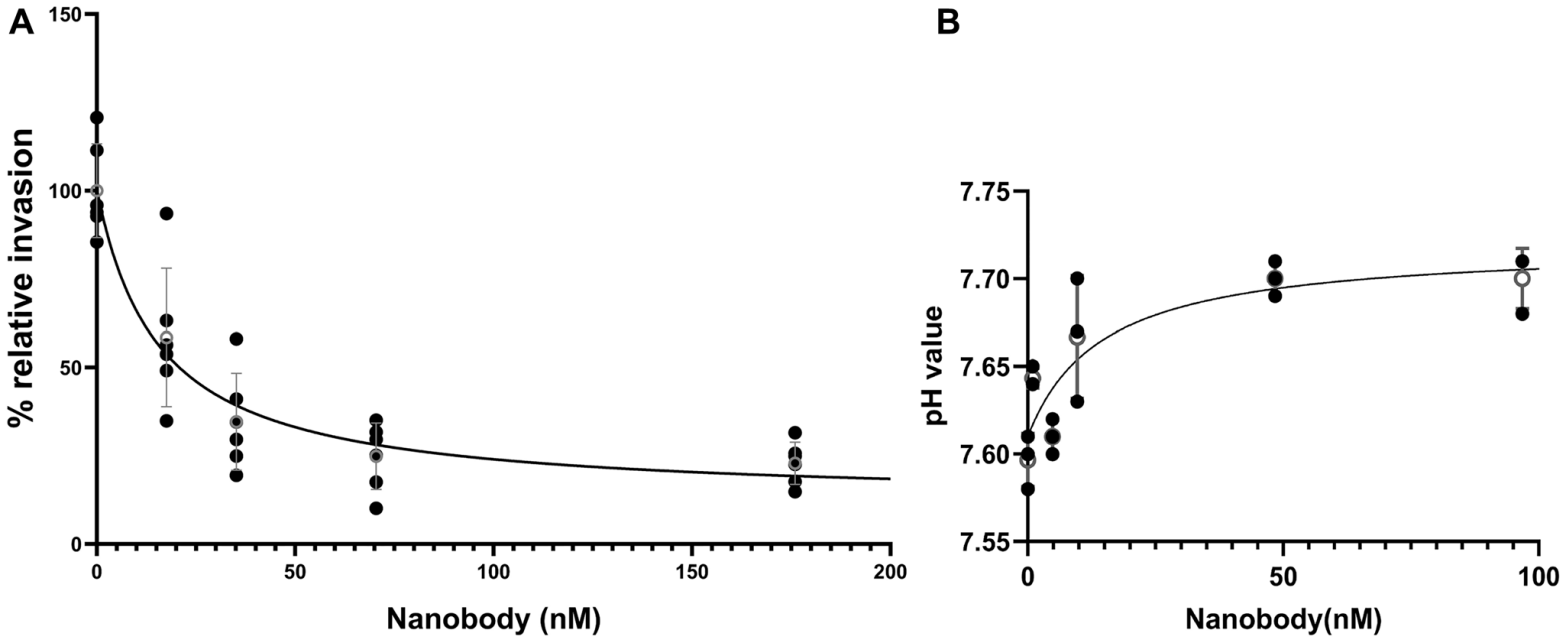
Dependence of *in vitro* invasion and extracellular acidification by 4T1-12B cells on the concentration of anti-V-ATPase nanobody. 4T1-12B cells were treated with the indicated concentrations of nanobody in PBS or PBS alone (**A**) *In vitro* invasion was measured by applying cells to Transwell inserts coated with Matrigel and induction of invasion by the presence of serum on the trans side of the well. Following incubation for 20 hrs, cells which had migrated to the trans side were stained with calcein and counted using a fluorescence microscope as described under Experimental Procedures. Shown are the combined results of two independent trials with three wells counted for each trial at each nanobody concentration. Error bars are SEM. The Ki value for the combined data is 15.6 (±5.3) nM (**B**) Extracellular acidification was measured for cells incubated with the indicated concentrations of nanobody in PBS (or PBS alone) for 2 hrs at 37°C followed by removal of the media and measurement of pH as indicated in the legend to Figure 2. Shown are the results of one representative trial. Error bars are SEM, open circle is the mean at each concentration. Mean Ki for the three independent trials is 11.6 (±1.5) nM.

To test whether the anti-V-ATPase nanobody can inhibit *in vitro* invasion of 4T1-12B cells, a transwell assay was performed as described under Experimental Procedures. Invasion was measured using Fluoroblock inserts coated with the extracellular matrix mimic Matrigel. Cells were applied to the cis side of the inserts and then induced to migrate to the trans side by the presence of serum as a chemoattractant. Cells that had migrated to the trans side were stained with calcein and counted using a fluorescence microscope. Fifteen separate wells were counted (three at each of five different nanobody concentrations) for each independent trial. As can be seen in Figure 4, treatment of cells with nanobody inhibited invasion with a similar affinity as inhibition of extracellular acidification. The Ki value for the combined trials of nanobody inhibition of invasion was 15.6 (±5.3) nM. The average Ki value for 3 independent trials of nanobody inhibition of extracellular acidification was 11.6 (±1.5) nM (the data shown is for one of the trials as it was not possible to combine the data from the three trials due to the different starting pH). These results indicate that the anti-V-ATPase nanobody inhibits both extracellular acidification and *in vitro* invasion of 4T1-12B cells with a similar affinity.

As a control, we used purified nanobody directed against GFP (see Experimental Procedures). Treatment of 4T1-12B cells with the anti-GFP nanobody had no effect on extracellular acidification or invasion at concentrations that maximally inhibited these processes for the anti-V-ATPase nanobody (0.6 μM anti-GFP nanobody showed invasion of 1.04 (±0.08) relative to control whereas 2.3 μM anti-GFP nanobody gave a delta pH of 0.01 (±0.01) relative to control).

### Administration of anti-V-ATPase nanobody inhibits metastasis of mammary fat pad-implanted 4T1-12B cells to lung in mice

**Figure 5:**
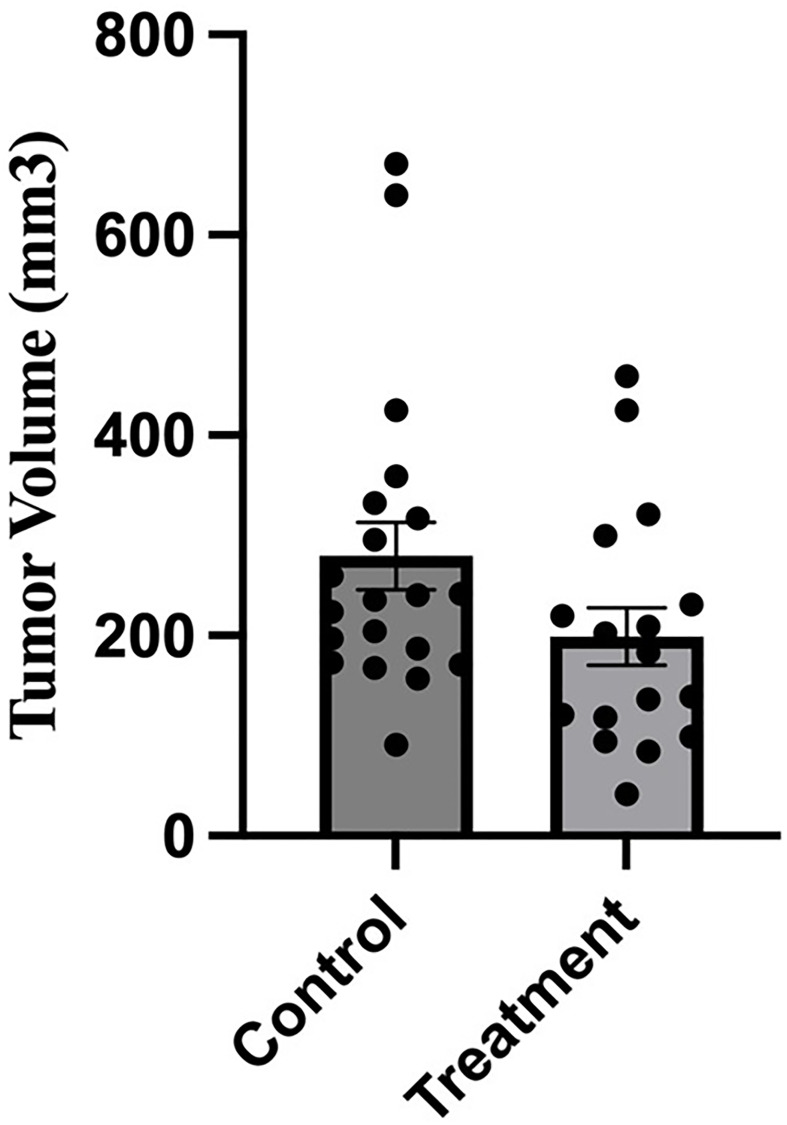
Administration of anti-V-ATPase nanobody does not affect growth of primary tumors in mice receiving mammary fat pad-implanted 4T1-12B cells. 20 BALB/c mice were implanted with 4T1-12B cells in the mammary fat pad and then received injections of 66 μg of nanobody in PBS (or PBS alone for control mice) IP three times per week for 3 weeks. Tumor volumes were measured prior to sacrifice by caliper measurement as described under Experimental Procedures. (*n* = 17 for treatment group, *n* = 20 for control group), (*P*-value = 0.08, error bars represent SEM).

We wished to test whether the inhibitory anti-V-ATPase nanobody altered tumor growth or metastasis following implantation of 4T1-12B cells in the mammary fat pad of mice. We first tested a range of amounts of nanobody (up to 66 ug) administered intraperitoneally to BALB/c mice three times per week for three weeks using 5 mice per group. This amount was chosen based upon the maximum dose tested for its effect on extracellular acidification (Figure 3). We found that none of the mice displayed any significant weight loss, reduction of viability or adverse health effects. We then proceeded to test the effect of nanobody administration on *in vivo* metastasis of implanted 4T1-12B cells. 1 × 10^6^ 4T1-12B cells in DMEM were implanted orthotopically into the intact no.4 mammary fat pad of 6 weeks old BALB/c mice. The mice were divided into two groups (20 mice per group), where the nanobody-treated group was injected with 200 μl PBS containing 66 μg of nanobody three times per week while the control group received an equivalent volume of PBS alone. Primary tumor volumes were determined using external caliper measurement three times per week for three weeks or until humane endpoint criteria were met. As shown in Figure 5, no statistically significant difference was observed in tumor volumes for the control and treatment groups at study endpoint.

**Figure 6:**
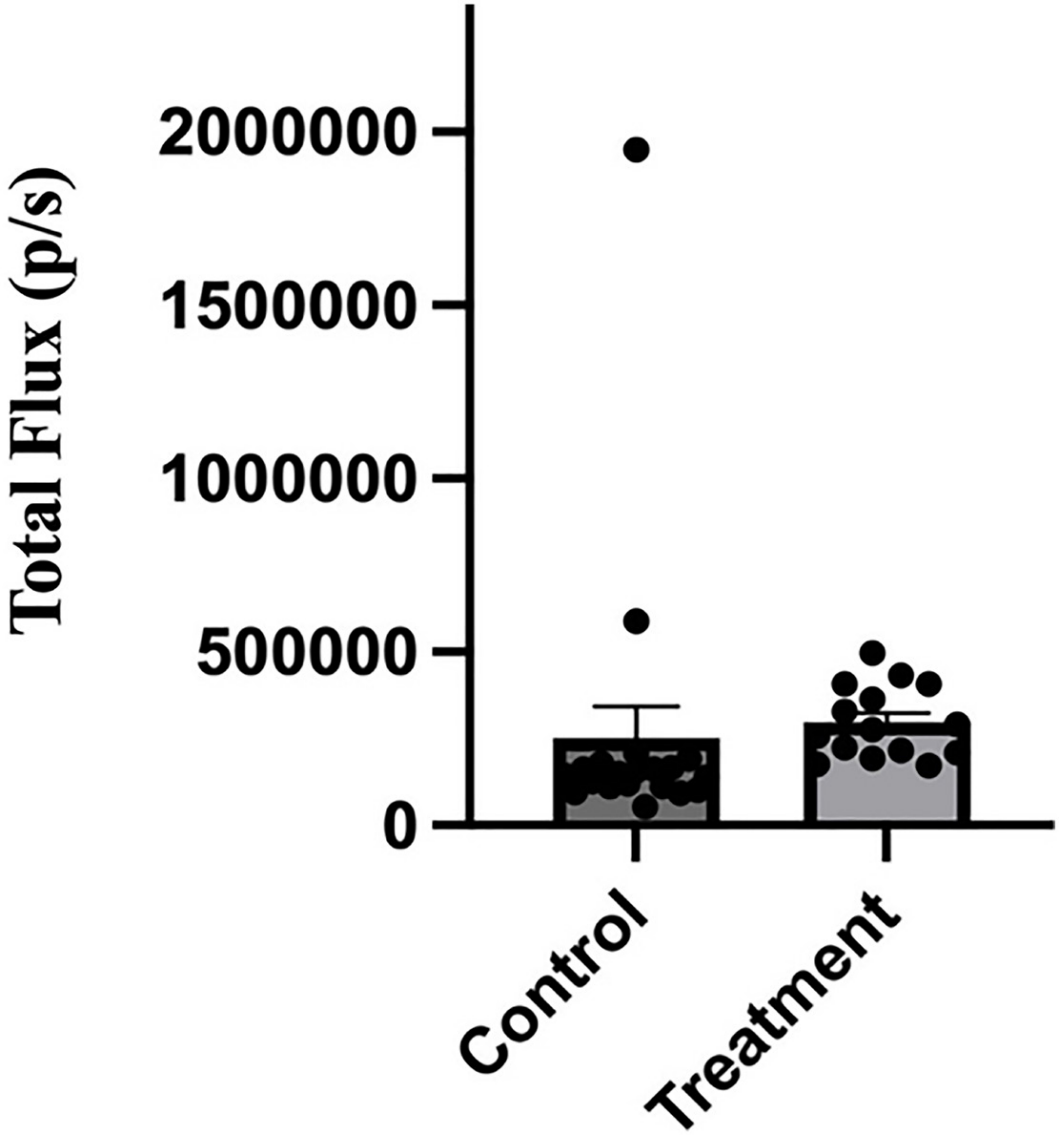
Administration of anti-V-ATPase nanobody does not affect intensity of leg metastases in mice receiving implanted 4T1-12B cells. 20 BALB/c mice were implanted with 4T1-12B cells in the mammary fat pad and then received injections of 66 μg of nanobody in PBS (or PBS alone for control mice) IP three times per week for 3 weeks. Prior to sacrifice mice were injected IP with luciferin and hind legs were removed and imaged *ex vivo* using a Perkin Elmer IVIS SpectrumCT *In Vivo* Imaging System. The intensity of the luminescence signal was quantitated using the Living Image^®^ software.

To determine whether nanobody treatment affected metastasis, mice at study endpoint were injected with luciferin and euthanized for *ex vivo* tissue imaging. Organs collected included spleen, liver, kidneys, heart, lungs, brain, and hind limbs. Organs were then imaged by biophotonic imaging using a Perkin Elmer IVIS SpectrumCT Imaging System. As summarized in Table 1, treatment of mice with the anti-V-ATPase nanobody reduced the frequency of lung metastasis from 50% (10 of 20 mice) to 13% (2 of 15 mice). Analysis using the chi-squared test indicates that these values are statistically different with a *p*-value of < 0.05. Two mice in the treatment group died on day 20 and one each on days 15 and 21 and an additional mouse was sacrificed due to reaching the humane endpoint on day 15. Thus only 15 of the 20 mice in the treatment group could be analyzed at study endpoint. No significant metastasis to other organs was observed in either the control or nanobody treated group, although all mice in both groups displayed bone metastases (Table 1). When the intensity of the bioluminescent signal of leg metastases in the control and treatment groups was compared, no significant difference was observed (Figure 6). As a control, a second group of mice receiving implanted 4T1-12B cells were injected with either PBS or 66 μg of the anti-GFP nanobody using the same regimen as described above. One of ten mice in the control group and three of ten mice in the anti-GFP-nanobody treated group died before study endpoint. Of the surviving mice, five of nine mice in the control group and four of seven mice in the anti-GFP-nanobody treated group developed lung metastases. The anti-GFP-nanobody treatment thus did not reduce lung metastases in mice in the same way that treatment with the anti-V-ATPase nanobody was observed to do. Thus, while treatment with the anti-V-ATPase nanobody had no significant effect on either tumor growth or leg metastases, a significant reduction in lung metastases was observed.

**Table 1:** Administration of anti-V-ATPase nanobody inhibits lung metastasis of implanted 4T1-12B cells in mice

Groups	Lung	Heart	Kidney	Spleen	Liver	Brain	Bone
Control	10/20	0/20	0/20	0/20	1/20	1/20	20/20
Nanobody	2/15	0/15	0/15	0/15	1/15	0/15	15/15

20 BALB/c mice were implanted with 4T1-12B cells in the mammary fat pad and then received injections of 66 μg of nanobody in PBS (or PBS alone for control mice) IP three times per week for 3 weeks. Prior to sacrifice mice were injected IP with luciferin and organs were removed and imaged *ex vivo* using a Perkin Elmer IVIS SpectrumCT *In Vivo* Imaging System. Organs were scored as positive or negative for metastasis based on luminescence signal intensity relative to background.

## DISCUSSION

Our previous results had demonstrated that selective inhibition of cell surface V-ATPases using either an antibody directed against an epitope tag introduced into an extracellular site on the c subunit or a membrane impermeant form of bafilomycin was able to inhibit invasion of MDA-MB231 human breast cancer cells *in vitro* [18]. These studies provided a proof of principal that inhibition of surface V-ATPases was a potential therapeutic approach to limiting breast cancer metastasis. It should be noted, however, that these studies employed a V-ATPase engineered to contain an extracellular V5-epitope tag in the c subunit. This was necessary because of the very limited mass of the V-ATPase exposed on the outside of the cell and the fact that the exposed sequences, including those in the native c subunit, are highly conserved and therefore difficult to prepare inhibitory antibodies against. To address this problem, we have prepared a nanobody against a native epitope expressed in the mouse c subunit that is exposed on the outside of the cell. The advantage of the nanobody approach is that, because a library of antibody sequences is screened *in vitro*, there is no selection against highly conserved self-antigens, as there would be in the traditional method of raising monoclonal antibodies in animals.

We have observed that the nanobody obtained by screening a camelid library against the mouse c subunit N-terminal sequence is able to inhibit cell surface V-ATPases in mouse 4T1-12B breast cancer cells. Cell surface V-ATPase activity was measured by measurement of extracellular acidification by cells suspended in unbuffered media (Figures 3, 4). While it is possible that nanobody binding to the extracellular side of the V-ATPase c subunit may directly inhibit activity through, for example, inhibition of rotary catalysis, it is also possible that crosslinking of V-ATPases at the cell surface may inhibit extracellular acidification by induction of their internalization via endocytosis.

We have previously shown that these cells employ the a4 isoform of the V-ATPase a subunit to target V-ATPases to the cell surface and that CRISPR/Cas9 disruption of the gene encoding a4 (but not the other a subunit isoforms) inhibits *in vitro* invasion as well as tumor growth and lung metastasis of implanted 4T1-12B cells [16]. Consistent with our previous results, we found that nanobody inhibition of plasma membrane V-ATPases in 4T1-12B cells led to inhibition of *in vitro* invasion of 4T1-12B cells (Figure 4). Moreover, the Ki values for nanobody inhibition of invasion and extracellular acidification are the same within the error of the measurements (Figure 4).

Consistent with the previously observed decrease in lung metastases upon a4 disruption (16), treatment of mice with the anti-V-ATPase nanobody also led to a significant reduction in lung metastases in mice receiving implanted 4T1-12B cells (Table 1). By contrast, no decrease in either tumor growth or leg metastasis was observed. It is possible that the lack of effect on tumor growth may be due to the inability of nanobody introduced intraperitoneally to adequately reach the primary tumor in order to inhibit its growth. This issue may be addressed by introducing the nanobody directly into the tumor by injection or by employing an alternate route of administration, such as intravenous injection. The lack of effect of nanobody treatment on leg metastasis is similar to the previously observed lack of decrease in the number of mice displaying leg metastases following a4 disruption (16). These results indicate that there may be differences in the mechanisms tumor cells employ to invade different tissues. For example, there may be differential roles of the V-ATPase in EMT [23], matrix metalloproteinase function [24] or regulation of kinase signaling [25]. It is also possible that nanobody availability is different for organs, such as lung, compared to peripheral sites, such as limbs, particularly when the nanobody is administered intraperitoneally. Further experiments testing the ability of nanobody to inhibit metastasis to different sites using alternative methods of administration should help to address this possibility.

Interestingly, in ovarian cancer cells, it appears to be the a2 isoform of the V-ATPase that targets the pump to the cell surface where it plays a role in cisplatin resistance [26]. Importantly, an antibody directed against a2 is able to slow the growth of ovarian tumors when administered to mice [27]. Because of the role of cell surface V-ATPases in avoiding apoptosis [28], inhibition of plasma membrane V-ATPases may also, in some cases, directly inhibit tumor cell growth and survival.

It is worth considering the potential side effects of an inhibitory, anti-V-ATPase nanobody directed against an extracellular epitope of the complex present on the surface of cancer cells when used as an anti-cancer therapeutic. A major benefit of this approach is that there are relatively few cells in humans that employ V-ATPases at the plasma membrane. Among these are renal alpha intercalated cells which utilize V-ATPases at the apical surface to secrete protons into the urine [29]. While the absence of these proton pumps leads to renal tubular acidosis [30], because they are present on the apical membrane of intercalated cells, they would not be affected by a membrane impermeant inhibitor such as a nanobody which was administered intravenously and would therefore be present in the blood. This is also true for a number of other cell types that utilize V-ATPases at the apical membrane, including the supporting and interdental cells in the inner ear, epithelial cells in the olfactory mucosa of the nose, β-cells in the islets of Langerhans and secretory duct cells in the pancreas, clear cells in the epididymis, microvascular endothelial cells in heart and lung, retinal pigment epithelial cells in the eye, ionocytes in the lung, clear cells in eccrine sweat ducts, and the acrosome of sperm cells [31–33]. Another major cell type which employs cell surface V-ATPases are osteoclasts, which utilize them for bone resorption [34]. An inhibitory nanobody present in the circulation would be expected to inhibit osteoclast function, which would inhibit bone resorption [35]. Because of the importance of osteoclast function in bone remodeling during development, inhibition of osteoclast V-ATPase activity could lead to developmental defects. In adults the primary function of osteoclasts is in calcium homeostasis, requiring careful monitoring of plasma calcium in patients treated with such inhibitors. A major benefit of inhibiting osteoclast function in patients with breast cancer, however, comes from the fact that breast cancer cells employ osteoclasts to invade bone [36]. By inhibiting osteoclast function, a further inhibition of breast cancer metastasis to bone may be achieved.

In conclusion, our results indicate that a nanobody directed against an extracellular epitope expressed on the surface of the V-ATPase is able to inhibit activity of cell surface V-ATPases in 4T1-12B breast cancer cells, inhibit *in vitro* invasion of these cells and inhibit metastasis of these cells to lung following their implantation in the mammary fat pad of mice. These results provide support for the use of an inhibitory antibody directed against an extracellular epitope of the V-ATPase as a potential anti-metastatic therapeutic to inhibit breast cancer metastasis.

## MATERIALS AND METHODS

### Materials and equipment

The following materials were obtained from the indicated companies: Nunc^™^ EasYDish^™^ Dishes 100mm (Thermo Fisher Sci#150464), BioCoat^™^ Matrigel^™^ Invasion Chamber with GFR Matrigel Matrix (Corning #354483), Falcon^™^ polystyrene 24-well microplates (Corning #353226), FluoroBlok^™^ 8.0 μm pore size insert (Corning #351152), Steriflip^™^ with 0.22 μm membrane (Sigma# SCGP00525), 0.20 μm sterile syringe filter (Corning #431222), dimethyl sulfoxide (Fisher #D128-500), calcein AM, 4mM in Anhydrous DMSO (Biotium #800111), DMEM with high glucose and pyruvate (Gibco #11995065), DMEM powder w/o glucose and L-glutamine (USBiological #D9800-02), Fetal Bovine Serum (Sigma #12306C), penicillin-streptomycin (Gibco #15140122), DMEM/F12 with HEPES (Gibco #11330032), human epidermal growth factor (Pepro Tech Inc #AF10015500UG), horse serum (Invitrogen #31874), hydrocortisone (Sigma #H0888), cholera toxin (Sigma #C8052), insulin (Sigma #I0516), trypsin-0.05% EDTA (Gibco #25300054), Bovine Serum Albumin (Sigma #A7906), 1× PBS pH 7.4 (Gibco #10010023), Concanamycin A (BVT-0237), Glucose (Sigma #G5400), L-Glutamine (Sigma #G5763), trypan blue (Sigma # T6146), XenoLight d-luciferin monopotassium salt (Perkin Elmer #122799). A Nikon Eclipse TE2000-S fluorescence microscope was employed to image calcein labeled cells. 4T1-12B cells ^1^ were a gift from Gary Sahagian [37] (Tufts University).

### Nanobody preparation

We have employed the company Proteogenix to prepare a synthetic nanobody directed against the N-terminal sequence of the mouse c subunit (MADIKNNPEY), which resides on the extracellular side of the membrane [38]. Since the c subunit is highly conserved among different species, we prepared a synthetic nanobody through *in vitro* screening of a phage display library. Single chain antibody sequences were derived from camelid. This approach permitted access to antibody sequences that would have been suppressed using conventional approaches to antibody preparation in mice or rabbits. This is because antibodies against self antigens (such as the highly conserved c subunit) are suppressed by the immune system. The immobilized peptide was conjugated to each of three different carriers (KLH, BSA and ovalbulin) for sequential rounds of biopanning. Panning was performed on a library of diverse (>10^10^) camelid single chain, variable heavy domain sequences fused to the phage coat protein [39]. After washing to remove non-binders, bound phage were eluted and amplified in bacteria. Biopanning was repeated for each of the carriers so that the highest affinity binders were enriched. Preliminary depletion of the library with each of the carriers alone was also performed before each biopanning step to reduce nonspecific binding. The highest affinity binders were expressed as chimeric proteins with the V_H_H domain fused to the mouse C_H_1 and hinge region together with a C-terminal His tag in CHO cells. The nanobody chimeras were then fused to generate dimeric nanobodies by disulfide bonding. This was so that the distance separating the antigen binding sites on the nanobody dimers was approximately the same as in IgG. The reason for generating bivalent nanobodies was that we previously used an IgG against V5 to inhibit V5-tagged V-ATPase [18]. Highest affinity binders were confirmed by ELISA and were purified with Nickle resin followed by SDS-PAGE analysis. Endotoxin removal was confirmed using a chromogenic LAL endotoxin assay kit.

### Anti-GFP nanobody purification

GFP nanobody in pOpine vector was a gift from Brett Collins (Addgene plasmid 49172 http://n2t.net/addgene:49172, RRID:Addgene_49172). The GFP nanobody (anti-GFPNb) was expressed in BL21DE3 cells grown to an OD_600_ ~0.5 and induced with 0.5 mM IPTG at 20°C for 20 h. Cells were harvested by centrifugation, resuspended in Buffer A (20 mM Tris, 250 mM NaCl, 10 mM Imidazole pH8) and frozen at –20°C until use.

For purification, cells were thawed in a room temperature water bath, DNase (80 μg/ml) and Lysoszyme (1 mg/ml) added and incubated 30 min on ice. PMSF was added (1 mM) before lysis by sonication 3 × 30 s each on ice followed by centrifugation at 13,000 × g, 40 min, 4°C. The supernatant was passed through a 0.45 μm filter before loading a 1 ml NiNTA column attached to an AKTA FPLC. After the initial binding step, the column was washed in 15 column volumes buffer A, followed by elution using a linear gradient to 60% buffer B (20 mM Tris, 250 mM NaCl, 500 Imidazole, pH 8). Pertinent fractions were pooled, concentrated using a ultrafiltration and applied to a Superdex 75 1.6 × 50 cm column in 20 mM Tris, 100 mM NaCl, pH 7.

For animal work, the purification scheme was the same with the following modifications. After loading onto the 1 ml NiNTA column attached to an AKTA FPLC, the column was washed sequentially in 5 CV buffer A, 5 CV buffer 2 (20 mM Tris, 250 mM NaCl, 1% Triton pH8), 5 CV buffer 3 (20 mM Tris, 250 mM NaCl, 25 mM sodium cholate pH8) to aid in the removal of Endotoxin. A final wash in 5 CV of buffer 1 led to release of the protein from the column. This fraction was concentrated and applied to a freshly scrubbed (1 CV water, 1 CV 0.5 M NaOH, 1 CV water) Superdex 75 1.6 × 40 cm column before dialysis overnight into PBS (137 mM NaCl, 27 mM KCl, 10 mM Na_2_HPO_4_, 1.8 mM KH_2_PO_4_, pH 7.4) at 4°C.

### Endotoxin removal from anti-GFP nanobody

For animal work, dialysate was applied to 1 ml Pierce High-capacity Endotoxin Removal resin (Thermo Scientific, 88276) and allowed to bind with rotation for 1 h at room temperature followed by 5 h at 4°C. Beads were pelleted by centrifugation, supernatant was collected and beads washed with 5 ml ET-free PBS, centrifuged and supernatant collected again. Supernatants were sterile filtered (0.2 μm) into sterile ET-free tubes and protein concentration determined using the extinction coefficient calculated using the Expasy Protparam server. Quantitation of Endotoxin levels was performed using the Pierce Chromogenic Endotoxin Quant kit (Thermo Scientific, A39552S) according to manufacturer’s instructions. Samples were used at 0.38 EU/ml.

### SDS-PAGE

Samples were boiled for 5 min at 95°C with 0.1% SDS and beta-mercaptoethanol before loading into a 12% Criterion^™^ TGX^™^ Precast Midi protein gel (Bio-Rad). The comb was gently removed and the gel was washed with distilled water or running buffer. The gel was placed into a Criterion tank according to manufacturer instructions and the chamber was filled with running buffer. The sample was loaded into the gel with a Hamilton syringe. Prestained Protein Ladder 10-250kDa Wide Range (BIO BASIC) was used. The electrophoresis was performed for 50 minutes at 200V. The gel was washed 3 times for 5 minutes in deionized distilled water. Bio-Safe^™^ Coomasie stain was added to the gel (enough to completely cover the gel). The gel was gently shaken for 1 h and then rinsed in deionized distilled water for at least 30 minutes. The gel was stored in water.

### Western blot

Following SDS-PAGE as described above, protein-containing gels were transferred to PVDF membrane (Bio-Rad) with the Trans-Blot Turbo transfer system (BioRad) before being blocked in 1% BSA in 0.1% TBS-Tween buffer for 30 min. After several washes with 0.1% Trs-buffered saline-Tween buffer, membranes were incubated with 1 μg/mL of rabbit anti-His antibody for 45 min for ECL visualization (Jacksonimmuno).

### Cell culture

The wildtype 4T1-12B cells used in this study were maintained in polystyrene-coated cell-culture dishes with DMEM that included high glucose and pyruvate, supplemented with 10% FBS and 1% penicillin/streptomycin (pen/strep). Cells were detached with trypsin-0.05% EDTA, and were cultured in a 5% CO_2_ humidified environment at 37°C.

### Transwell invasion assay

*In vitro* transwell invasion assays were performed as follows. Transwell inserts were first washed with PBS and then coated with 10 ug of Matrigel followed by incubation for 2 hrs at 37^o^C to allow polymerization of the Matrigel. Matrigel-coated inserts were then placed into a new well containing 750 μl of DMEM with 10% FBS. Cells were trypsinized and resuspended to a final concentration of 3 × 10^5^ cells/ml in DMEM containing 0.1% BSA. Where indicated, either 0.1% DMSO, the indicated amounts of concanamycin A in DMSO, PBS or the indicated amounts of nanobody in PBS were added to the cell suspension. 500 μl of the cell suspension was added onto each Matrigel insert. Each treatment was done in triplicate wells. Cells were then incubated at 37°C for 20 h, The inserts were placed into wells containing 4 ug/ml calceinAM in PBS and incubated for 20 min at 37°C in 5% CO_2_. Cells that had invaded to the trans side were imaged using a Nikon fluorescence microscope. An average of 8 images were taken per well, and the number of invading cells was calculated for each of three wells under each condition.

### Extracellular environment pH analysis

1 × 10^5^ 4T1-12B cells were suspended in 1 ml of DMEM media to which was added either PBS or the indicated amounts of nanobody in PBS followed by incubation for 2 hrs at 37°C to allow time for the nanobody to bind to its cell surface target. The media was then replaced with 1 ml of unbuffered DMEM containing either 0.1% DMSO plus 1:5 diluted PBS (control), 1:5 diluted PBS plus concanamycin in DMSO to the indicated final concentration (conA) or 0.1% DMSO plus 1:5 diluted PBS containing the indicated amounts of nanobody (nanobody) followed by incubation for an additional 2 hrs at 37°C. Where indicated, PBS was diluted 1:5 with DMEM. The pH of the media was then measured using a Beckman pH meter, with each measurement done in triplicate.

### *In vivo* nanobody toxicity study

Twenty wild type female BALB/C mice were randomly assigned to four different dosage groups. Each group received the same volume (200 μl) of either PBS alone, or PBS containing 16.5 μg, 33 μg and 66 μg of nanobody. Administration was three times per week intraperitoneally over a period of 3-weeks, with the mice followed for an additional week after the final dose. The maximum dose employed for this study was selected based upon our *in vitro* results (Figure 3).

### *In vivo* metastasis model

Wild type 4T1-12B cells were seeded at 1×10^5^/cm^2^ (approximately 40% confluency) and allowed to attach overnight. The following day cells had reached approximately 80% confluency and were detached by trypsinization and verified to be ≥95% viable by trypan blue exclusion. Cells were then centrifuged at 300×*g* for 5 minutes, and the cell pellets were resuspended in fresh DMEM at 1 × 10^7^ cells/ml. 100 μl of the resulting cell suspension were injected into the intact no. 4 mammary fat pads of 6-week-old female BALB/c mice using a 26-gauge needle. Primary tumor dimensions were acquired prior to sacrifice by caliper measurement, and tumor volume was calculated using the modified ellipsoid formula (L × W^2^)/2 [40].

### Bioluminescent imaging

*In vivo* imaging was performed on the first day post-implantation to verify accurate implantation of cells. A fresh luciferin solution was prepared by dissolving XenoLight d-luciferin monopotassium salt (Perkin Elmer #122799) in PBS at 10 mg/ml. The luciferin solution was filter-sterilized, and 100 μl was injected into each mouse intraperitoneally. Mice were anesthetized with a 2.5%/97.5% isoflurane/O_2_ mixture using a Caliper Life Sciences XGI-8 Gas Anesthesia System, and imaged 10 minutes post-luciferin injection using a Perkin Elmer IVIS SpectrumCT *In Vivo* Imaging System. At the conclusion of the study (or when humane endpoints were reached), animals were sacrificed, and organs were removed and imaged *ex vivo* following the same luciferin injection protocol. Because the intensity of the signal from the primary tumor prevented detection of metastases in the intact mice, live animal imaging was not performed after the initial imaging described.

### Animal care

40 Female BALB/c mice aged 6 weeks were purchased from The Jackson Laboratory (Bar Harbor, ME), and housed within the Tufts University animal facility. All animal work was approved by and carried out in accordance with the Tufts University Institutional Animal Care and Use Committee. Mice which reached the humane endpoint (a tumor volume greater than 1500 mm^3^ or a tumor ulceration diameter larger than 7 mm) were euthanized. One mouse in the treatment group was observed with foot necrosis and was euthanized for humane reasons. No mice were found to have suffered a loss in body weight ≥15% from baseline. Four mice in the treatment group were found dead at week 2.

### Statistics

Analysis using an unpaired two-tailed *t*-test with Welch’s correction was performed to compare treatment to control groups. Statistical tests were considered significant at *p* < 0.05. Error bars represent SEM. *In vivo* lung metastasis frequency between control and treatment groups was compared using the chi-squared test and shown to be significantly different with a *p*-value of < 0.05.

## Abbreviations

BSA: Bovine serum albumin
CHO cells: Chinese Hamster Ovary cells
ConA: Concanamycin A
CV: Column volumes
DMEM: Dulbecco’s modified Eagle medium
DMSO: Dimethyl sulfoxide
ECM: Extracellular matrix
EMT: Epithelial-mesenchymal transition
ET: Endotoxin
GFP: Green fluorescent protein
IP: Intraperitoneal
IPTG: Isopropyl ß-D-1-thiogalactopyranoside
KLH: Keyhole limpet hemocyanin
PBS: Phosphate buffered saline
Pen/strep: Penicillin/streptomycin
PMSF: Phenylmethylsulfonyl fluoride
siRNA: Small interfering ribonucleic acid
V0: V-ATPase membrane domain
V1: V-ATPase peripheral domain
V-ATPase: Vacuolar H^+^-ATPase
V_H_H: Variable domain of heavy chain-only antibody, also known as nanobody
